# How does political discussion frequency impact political moral opinions? The moral argument theory of opinion dynamics

**DOI:** 10.3389/fpsyg.2022.915252

**Published:** 2022-09-02

**Authors:** Kimmo Eriksson, Irina Vartanova, Pontus Strimling

**Affiliations:** ^1^Institute for Futures Studies, Stockholm, Sweden; ^2^School for Education, Culture and Communication, Mälardalen University, Västerås, Sweden

**Keywords:** political discussion, public opinion, liberalization, polarization, moral argument theory

## Abstract

Discussions of political issues may influence people's opinions. Is there any systematic difference in opinions between those who discuss frequently and those who do not? We measured the association between self-reported discussion frequency and the probability of holding the more liberal opinion on moral issues, using data from the General Social Survey (81 issues, *n* = 4,395) and the American National Election Studies (27 issues, *n* = 17,653). This association looked different among liberals and among conservatives. Having more frequent discussions is associated with a higher probability of holding more liberal opinions among liberals, while there is little association between discussion frequency and opinions among conservatives. These findings can be explained by the moral argument theory, which is an account of the long-term liberalization of public opinion on moral issues as an outcome of repeated discussions. The key assumption of this theory is that opinions that are justified by the kinds of arguments that only conservatives accept have a disadvantage compared to opinions that are justified by the kinds of arguments that everyone accepts. Consistent with this theory, we find that the effect of discussion frequency is stronger for moral opinions that have a bigger argument advantage.

## Introduction

Political discussion is an important form of political communication. People's opinions tend to be influenced by the views of their discussion partners (Pattie and Johnston, 2001). This raises the possibility that people's opinions might show some association with the frequency with which they discuss political issues. While prior research has linked the frequency with which individuals discuss political issues to their political participation, political knowledge, argument repertoire, and accuracy of beliefs (Price et al., 2002; Eveland, 2004; Eveland and Hively, 2009; Amsalem and Nir, 2021), we are not aware of any research addressing the relationship between discussion frequency and opinions. In the present paper, we will demonstrate empirically that this relationship looks different among liberals than among conservatives. Among liberals, those who discuss more frequently are more likely to have more liberal moral opinions. Among conservatives, by contrast, there is little systematic effect of discussion frequency on opinions.

This empirical pattern could have a number of explanations. Here we focus on how it can be understood in terms of the moral argument theory of opinion dynamics (Eriksson and Strimling, 2015). This theory makes a unique and testable prediction about which moral issues will exhibit the largest effect of discussion frequency among liberals.

### The moral argument theory of opinion dynamics

The moral argument theory of opinion dynamic was designed to explain why public opinion on moral issues tends to become more liberal over time (Eriksson and Strimling, 2015). This theory is based on four assumptions:

1. In their daily life, people will often be taking part in, or observing, discussions of moral issues that will expose them to arguments for different opinions. Such exposure is evidenced by media content analyses (Clifford and Jerit, 2013; Clifford et al., 2015).
2. Moral arguments based on harm, violence, fairness and liberty (HVFL) are generally acceptable, that is, relevant to the moral judgments of both liberals and conservatives; other kinds of moral arguments (e.g., purity, loyalty, authority, government overreach) are relevant mainly to conservatives and therefore not generally acceptable. This assumption is supported by extensive studies asking liberals and conservatives how relevant different kinds of arguments are for their moral judgments (Graham et al., 2009, 2011; Eriksson et al., 2022).
3. For any specific moral opinion, there is general agreement that only certain kinds of moral arguments can be used to justify it. On any given issue, one opinion will therefore have a “HVFL argument advantage” in the sense that this opinion is easier to justify by the generally acceptable HVFL kinds of arguments than the opposite opinion is. Studies conducted in the United States, the United Kingdom, Brazil, and Israel have demonstrated that measures of which arguments justify which opinions are virtually identical across different groups, such as across liberals and conservatives (Vartanova et al., 2021). Thus, these measures reflect genuine connections between opinions and different kinds of arguments, the perceptions of which are largely independent of what opinion people hold (Strimling et al., 2019).
4. When confronted with an argument, people are more likely to change opinion if the argument is of a kind that is relevant to their moral judgments. This is supported by experimental studies (Jansson and Strimling, 2022).

Taken together, assumptions 2 through 4 say that when people are exposed to arguments, opinion switches are more likely to be toward the opinion with HVFL argument advantage than to the opposite opinion, especially among liberals (Eriksson and Strimling, 2015). At the population level, this mechanism is expected to generate opinion dynamics in which the opinion with HVFL argument advantage gradually becomes more popular, first among liberals and then also among conservatives (Strimling et al., 2019).

The validity of the theory is supported by recent studies showing that measures of the HVFL argument advantage of opinions can predict several important things about public opinion. First, whether an opinion will be more popular among liberals or among conservatives is extremely accurately predicted by whether the opinion is advantaged or disadvantaged with respect to HVFL arguments, respectively (Strimling et al., 2019). Second, opinion trend data show that opinions with HVFL argument advantage tend to become more popular over time and at a speed that is proportional to the size of the advantage (Eriksson et al., 2022). The HVFL argument advantage has even been used to make accurate predictions about future opinion changes, that is, from one wave of an opinion poll to the next wave several years later (Strimling et al., 2022). The success at predicting future opinion change constitutes strong evidence for the causal claim that the HVFL advantage of an opinion plays a direct role in the process that creates long-term change in public opinion.

Here we develop the theory further by taking individual differences in discussion frequency into account. The aim is to derive testable predictions of how individuals' discussion frequency is associated with the probability that they hold certain moral opinions.

### A computational model

To derive predictions from the moral argument theory, prior research has used a computational model (Strimling et al., 2019). Here we extend this model by incorporating individual differences in discussion frequency. To do so we must make additional assumptions about whether those who have frequent discussions and those who have less frequent discussions tend to discuss with different people. It is likely that people who discuss politics frequently tend to have discussion partners that they agree with, as prior research indicates that discussions between those agree are more frequent than discussions between those who disagree (Mutz, 2006; Morey et al., 2012; Eveland et al., 2018). In the model, we operationalize this assumption by setting the probability that an agent discusses with someone of the agent's own ideological group (liberals or conservatives) as higher for agents that discuss with a higher frequency. We then examine that the main model predictions are independent of the strength of this bias.

**Figure 1:**
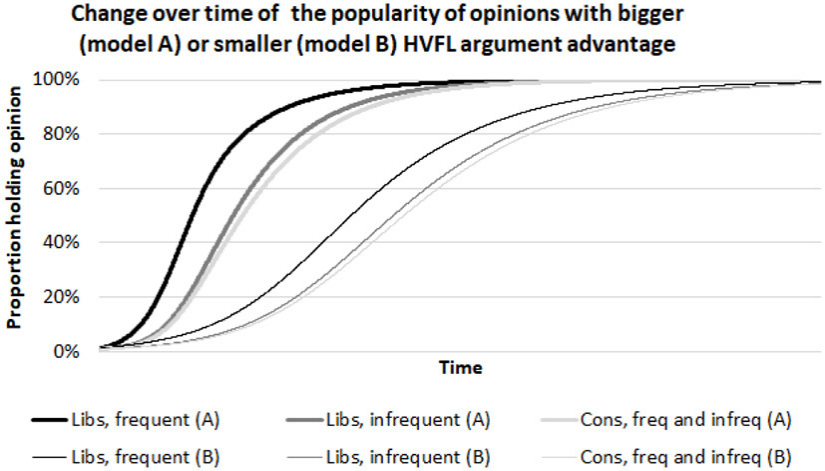
Typical runs of the computational model differing only in the HVFL argument advantage set to be bigger in Model A and smaller in Model B. On the x-axis is time, on the y-axis is the popularity of an opinion with HVFL argument advantage. For details, see the Supplementary material.

The model describes a social dynamical process in which liberal and conservative agents in a population meet and argue for their opinion and sometimes change their opinion in accordance with the assumptions of the moral argument theory. Details of the set-up and analysis of the computational model is provided in the Supplementary material. Figure 1 shows two typical runs of the model, differing only in the size of the HVFL argument advantage (bigger in Model A, smaller in Model B). The first thing to note about these simulations is that the popularity of the advantaged opinion is always higher among liberals than among conservatives. In line with this model prediction, an empirical study in the United States found an extremely strong correlation between which opinions are more popular among liberals and which opinions have argument advantage (Strimling et al., 2019). We shall test that this finding is replicated in the data used in the current study (Prediction 1).

The second thing to note in Figure 1 is that the popularity of advantaged opinions increases over time and that the rate of the increase is higher for the opinion with the bigger advantage. In other words, the model predicts that the HVFL argument advantage of an opinion determines both the direction and speed of change in public opinion. Empirical studies have verified this prediction in both the United States and the United Kingdom (Strimling et al., 2019; Eriksson et al., 2022). We shall test that this finding too is replicated in the data used in the current study (Prediction 2).

We now turn to the novel aspect of the model, which is the role played by individual differences in discussion frequency. In Figure 1, the popularity of the advantaged opinion is higher among frequently discussing liberals than among infrequently discussing liberals. In other words, the model predicts a positive effect of discussion frequency on the probability that a liberal holds an advantaged opinion (Prediction 3). To see why the model produces this effect, consider that we assume that liberals who take part in a discussion are more likely to change their opinion toward the opinion with HVFL argument advantage than in the opposite direction (this is what this causes their upward trend in Figure 1). The probability that this opinion change has occurred at a given point in time is higher for a liberal who takes part in discussions more frequently.

Next, Figure 1 illustrates that the effect of discussion frequency among liberals is stronger for the opinion with bigger HVFL argument advantage (Prediction 4). The model produces this prediction because opinion change among liberals comes about precisely from the combination of their taking part in discussions and the opinion's HVFL argument advantage.

Finally, we turn to conservatives. Figure 1 shows zero difference in opinions between frequently and infrequently discussing conservatives. In other words, the model predicts no effect of discussion frequency among conservatives (Prediction 5). Actually, this prediction relies on a certain assumption on the extent of ideological ingroup bias in high-frequency discussions. If this assumption is relaxed, the model may produce a small effect of discussion frequency that favors either the advantaged or the disadvantaged opinion, but the effect among conservatives is always smaller than the effect among liberals (Models C and D in the Supplementary material). To see why, consider that the model assumes conservatives are equally influenced by HVFL arguments and other kinds of arguments. This means that discussion among conservatives yield no net effect on opinions. The systematic opinion change of conservatives that we see in Figure 1 arises because they are assumed sometimes to discuss with liberals. Whether it is frequently or infrequently discussing conservatives that are more likely to hold the advantaged opinion will therefore depend on the exact assumption about how often each group discusses with liberals.

### An empirical test of the model predictions

We have outlined five predictions produced by a computational model based on the moral argument theory. To empirically test these predictions, we need to estimate the effect of discussion frequency on the probability of holding opinions with HFVL argument advantage. We achieve this by analyzing data on people's self-reported discussion frequency, their ideological affiliation, and their opinions on multiple moral issues, obtained from the American National Election Studies and the General Social Survey. For the issues covered by the General Social Survey, measures of the HVFL argument advantage are obtained from a previous study (Eriksson et al., 2022). For the issues covered by the American National Election Studies, we here collect new data on the HVFL argument advantage.

## Methods

### Measures from the American National Election Studies

The American National Election Studies is a nationally representative sample survey of electoral behavior, political participation, and public opinion in the US population. It has been conducted in connection with national elections since 1948. Data is usually gathered from in-person interviewing. The number of completed interviews in a given year ranges between 1,200 and 2,500. To obtain representativity, the American National Election Studies makes use of complex probability sampling. We used data from the American National Election Studies up to 2016. The 2020 wave is excluded because it did not include the media use variable we use as a control.

#### Ideological affiliation

The American National Election Studies includes a measure of liberal-conservative self-identification on a 7-point scale from extremely liberal to extremely conservative. To obtain more robust estimates of the effect of discussion frequency among liberals and conservatives, we collapse this scale to a ternary categorization into liberals (steps 1 through 3; 27%), moderates (step 4; 33%), and conservatives (steps 5 through 7; 40%). Estimates for each of the seven steps are reported in Supplementary Figure 1.

#### Political discussion frequency

Since 1984, the American National Election Studies has measured political discussion frequency using the item “How many days in the past week did you talk about politics with your family or friends?”.^1^

#### Moral opinions

From the set of all items that had been asked in at least three waves of the American National Election Studies, research assistants identified items covering opinions on moral issues, that is, questions about right and wrong in a non-economic sense (Strimling et al., 2019). The final selection included 27 items on issues such as abortion, sexual behaviors, civil rights, gun rights, and the death penalty. For a full list, see Supplementary Table 1. Some of the selected items include neutral responses (such as “neither agree nor disagree”) and/or graded responses (such as “slightly agree” and “strongly agree”). To make analyses comparable across items, we dichotomized all items by omitting neutral responses and by combining graded responses, following prior research (Strimling et al., 2019; Eriksson et al., 2022). The dataset comprised more than 280,000 data points on moral opinions from 17,653 respondents. Thus, for the average respondent we have opinion data on 15.6 (out of 27) items. The number of data points per item varied between 1,806 and 18,633, reflecting the number of waves in which an item had been included.

### Measures from the general social survey

The General Social Survey is a survey of behaviors and attitudes in the US population (Smith et al., 2019). The survey has been conducted since 1972 and became biennial in 1994. The General Social Survey makes use of computer-assisted personal interviews and a multistage probability sampling design to gather data pertaining to non-institutionalized adults who are at least 18 years old. Four waves of the General Social Survey (1985, 1987, 2000, and 2014) included some measure of political discussion frequency together with a measure of political ideology. The response rates for each of these waves were 78.7, 75.4, 70.0, and 69.2%, respectively. This data is available for a total of 4395 respondents.

#### Political ideology

The General Social Survey uses the same measure of self-reported ideology as the American National Election Studies. Using the same ternary categorization as in Study 1, the dataset includes 27.2% liberals, 39.3% moderates, and 33.4% conservatives.

#### Political discussion frequency

Political discussion frequency has been measured using different items across different waves of the General Social Survey. For example, the 2014 wave included the item “When you get together with your friends, relatives or fellow workers, how often do you discuss politics?”, with responses on a four-step scale ranging between never and often. In the 2000 wave the focus was on discussions in the last year: “In the last 12 months, have you discussed your views about political or social policy issues, current affairs, or political campaigns with other people?” (Not at all; 1 or 2 times; 3 or more times). The 1985 and 1987 waves focused on discussions with the respondent's most important discussion partners: “From time to time, most people discuss important matters with other people. Looking back over the last six months - who are the people with whom you discussed matters important to you? [..].” The 1985 question then read “Thinking of the people/person we've just been discussing, would you say you discuss social and political issues with them almost all the time, most of the time, occasionally, or almost never?”, while the 1987 question read “About how often do you talk to (NAME) about political matters?” (Almost daily; At least weekly; At least monthly; At least yearly; Less than yearly).

#### Moral opinions

From the General Social Survey items included in any of the six aforementioned waves, we selected 81 items previously identified as opinions on moral issues (Vartanova et al., 2021). A full list is provided in Supplementary Table 2. Items were dichotomized as described above for the American National Election Studies.

### Measures of the HVFL argument advantage of moral opinions

Measures of the HVFL argument advantage of each of the 81 moral opinions in the General Social Survey were derived by Eriksson et al. (2022), based on data from Vartanova et al. (2021). Following the same procedure, we conducted a new data collection to obtain measures of the HVFL argument advantage of the 27 moral opinions in the American National Election Studies. In accordance with Swedish legislation and institutional requirements, ethical review and approval was not required for this fully anonymous survey study.

#### Participants

Two hundred and fifteen participants (52 % females, mean age 42.3 years, SD = 11.2) were recruited among users of Amazon Mechanical Turk. After giving informed consent, participants were presented with a series of moral opinions drawn in random order from the 27 items in batches of 9. The participant could choose whether to judge one batch or two, or even all three batches; the average participant judged 12.3 items and the average item was judged by 98 participants. Amazon Mechanical Turk internal prescreening was used so that each item was judged by an approximately equal number of liberals and conservatives.

#### The pool of moral arguments

We used a pool of arguments from Vartanova et al. (2021), adapted from the Moral Foundations Questionnaire (Graham et al., 2011). The pool is based on a categorization of moral arguments (fairness, harm, violence, liberty, authority, loyalty, and purity), each of which is represented by three arguments in the pool. For example, the three fairness arguments are “someone is denied his or her rights,” “someone acts unfairly,” and “some people are treated differently from others.” For a full list, see Supplementary Table 3.

#### Procedure

Participants were presented with one item at a time (e.g., “By law, prayer should not be allowed in public schools.”). After providing their answer, using a dichotomous response scale (yes/no), participants were asked to consider why they chose that answer. Specifically, they were presented with a list consisting of a random draw of one argument of each kind from the pool of arguments (plus “some other reason”) and asked to tick all arguments that apply. Arguments were worded to match whether the participant had answered yes or no (e.g., “Yes, because otherwise someone is denied his or her rights” or “No, because then someone is denied his or her rights”). Participants were then asked for the arguments they expected to be chosen by someone who had given the *opposite* answer to the item. The same selection of arguments, but reworded to match the opposite answer, was presented for the participant to choose from. Thus, every participant chose arguments for both sides on the issue.

#### Calculation of HVFL argument advantage measures

To calculate the HVFL argument advantage for an item we focus on harm, violence, fairness, liberty. Data on these four kinds of arguments for each side (yes/no) of a moral issue were coded as eight dummy variables; e.g., the dummy variable *yes:fairness* was coded 1 if the participant judged the fairness argument to apply to the “yes” opinion on the item, 0 otherwise. For each participant, a measure of the argument advantage for the item was then obtained as (*yes:harm* + *yes:violence* + *yes:fairness* + *yes:liberty*)/4 – (*no:harm* + *no:violence* + *no:fairness* + *no:liberty*)/4. For example, say that a participant said that the “yes” opinion could be justified by a fairness argument and a harm argument, while the “no” opinion could be justified by a liberty argument. The argument advantage of the “yes” opinion would then be ½ – ¼ = 0.25. The theoretical range is from −1 to 1.

For each item we estimate the HVFL argument advantage in the population, by averaging the argument advantage measure over all participants who had judged the item. Estimates for all items are reported in Supplementary Table 1, ranging from −0.24 to 0.28 (M = 0.00, SD = 0.16). Compared to this variation, the standard errors of the estimates were small (ranging from 0.02 to 0.04). In other words, the variation across items is genuine and not an artifact of sampling errors. We also replicate the finding that HVFL advantage measures are essentially independent of the ideology of the respondents (Vartanova et al., 2021); the Pearson correlation is extremely high, 0.92, between HVFL advantage measures based on liberals and the same measures based on conservatives. We conclude that these measures of argument advantage reflect meaningful differences between moral opinions in how well they can be justified by generally accepted moral arguments.

### Analysis

#### Estimating opinion change rates in the American National Election Studies data

Opinion change rates were estimated using logistic regression with time, measured in decades, as predictor, yielding change rates in terms of change in log odds per 10 years. The sign of the change rate tells us whether the opinion has increased or decreased in popularity.

#### Estimating effects of discussion frequency on opinions

We estimate the effect of political discussion frequency upon the probability of holding the advantaged opinion using logistic regression of dichotomized opinion measures, coded so that 1 refers to the opinion with HVFL argument advantage on the issue in question, 0 refers to the opposite opinion. As discussion frequency may be confounded with other demographic variables that are associated with opinions, we control for education, gender, age, ethnicity, and the year the survey was conducted. Datasets and inclusion of random effects depend on the aim of the analysis as follows.

To estimate the discussion frequency effect on opinions on a given issue (e.g., whether the government should see to it that white and black children go to the same schools) in a given group (e.g., among liberals), we use only the data on that issue from that group. No random effects are included. Thus, the model specification is simply


$$\begin{array}{l} logit\left(Opinion_{i}\right)\;=\;\beta_{0}\;+\;\beta_{1}DF_{i}\;+\;\beta X_{i}, \end{array}$$

where *Opinion*_*i*_ is the opinion of individual *i, DF*_*i*_ is the political discussion frequency of individual *i*, and *X*_*i*_ are the control variables: education in years, gender (dummy variable for woman), age in years, ethnicity status (dummy variables for black and other, compared to white), and media use (in the General Social Survey we use the item “How often do you read the newspaper—every day, a few times a week, once a week, less than once a week, or never?”; in the American National Election Studies we use the maximum of “How many days in the past week did you read a daily newspaper?” and “How many days in the past week did you watch national news on TV?”). All continuous variables are standardized with zero mean and unit standard deviation. In the General Social Survey the DF effects are estimated in a given wave of data collection (e.g., the 1985 General Social Survey), but the American National Election Studies includes the same DF and opinion measures in multiple waves so for analyses of data from the American National Election Studies we additionally control for year. Coefficient β_1_ is the discussion frequency effect in the unit of log odds. By exponentiating β_1_ we obtain the factor by which the odds for holding the advantaged opinion on the given issue increases with an increase in discussion frequency by one standard deviation.

Due to limited sample sizes, estimates of the discussion frequency effect per issue will not be very reliable. We use them only for illustrative purposes. To test the prediction of a positive discussion frequency effect among liberals, we instead pool the data from that group across all issues and waves and estimate the average discussion frequency effect. To account for non-independence of data from the same individual (*i*), or data on the same issue-wave (*j*), or data from the same issue(*k*) we include random intercepts for each individual (*u*__*i*_00_), issue-wave (*u*_0j*k*_), and issue (*u*_00k_). The formal model specification is


$$\begin{array}{l} logit\left(Opinion_{ijk}\right)\;=\;\left(\beta_{0}\;{+u_{i}}_{00}\;{+u}_{0\text{j}k}\;{+u}_{00\text{k}}\right)\;+\;\beta_{1}DF_{i}\;+\;\beta X_{i.} \end{array}$$

To test the prediction about a moderating effect of the HVFL argument advantage of the issue, we extend the model by including also the HVFL argument advantage (centered at the mean and multiplied by 10 so that 1 unit corresponds to 0.1 in the original HVFL advantage score) and its interaction with discussion frequency:


$$\begin{array}{l} logit\left(Opinion_{ijk}\right)\;=\;\left(\beta_{0}\;{+u_{i}}_{00}\;{+u}_{0\text{j}k}\;{+u}_{00\text{k}}\right)\;+\;\beta_{1}DF_{i}\;+\\ \beta_{2}HVFL_{i}\;+\;\beta_{3}DF_{i}\times HVFL_{i}+\;\beta X_{i} \end{array}$$

Here, coefficient β_3_ measures how the effect shifts with a change of one unit in the HVFL argument advantage.

We use the lme4 package version 1.1-27.1 (Bates et al., 2015) in R to estimate the models by maximum likelihood. Survey weights provided by the American National Election Studies were accommodated in the estimation to account for unequal sampling probabilities.

## Results

### Prediction 1: HVFL argument advantaged opinions are more popular among liberals than among conservatives

**Figure 2:**
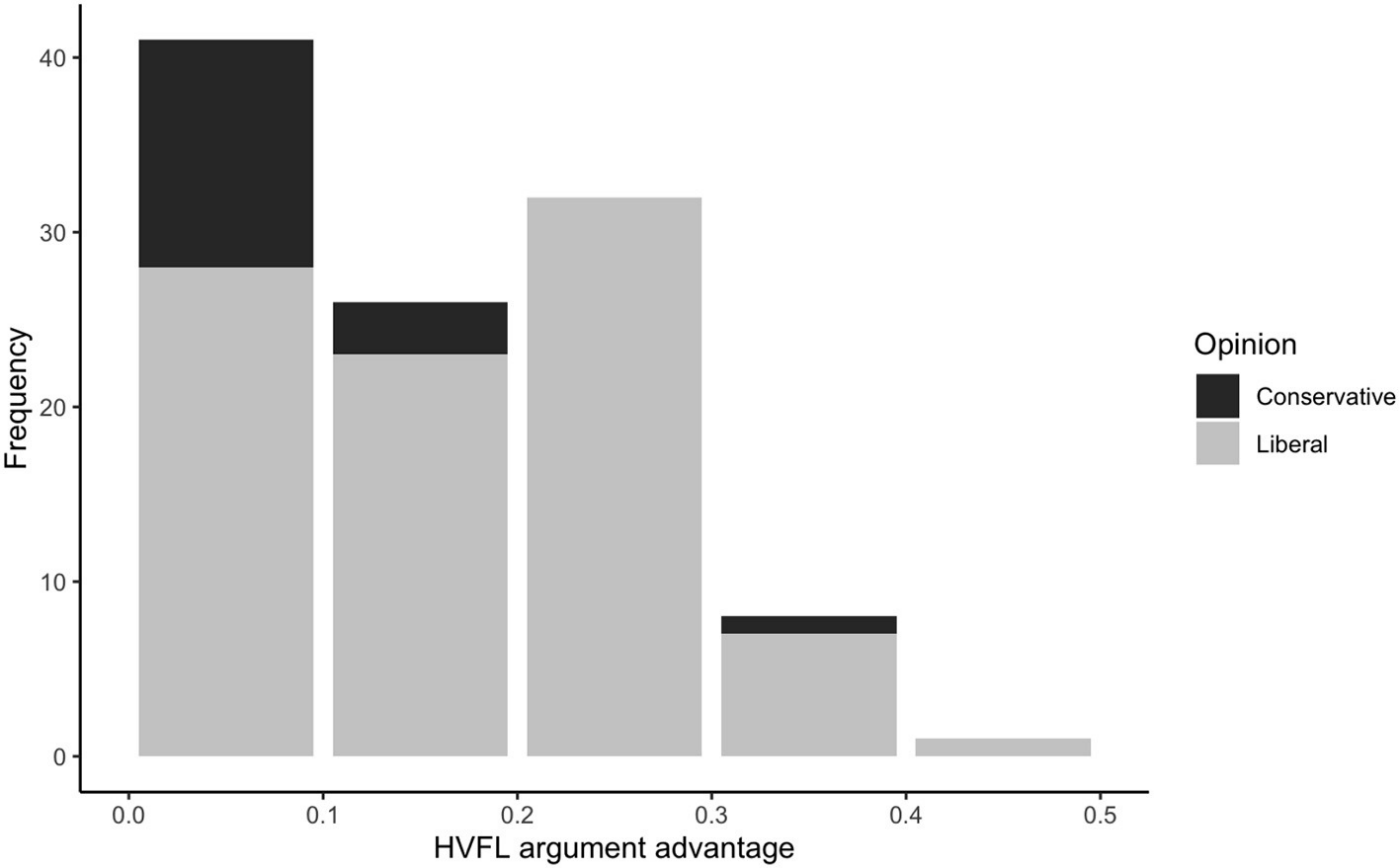
The distribution of the HVFL argument advantage for 27 moral opinions in the American National Election Studies and 81 moral opinions in the General Social Survey (reverse coded in case the stated opinion was disadvantaged). Each bar of the histogram shows the number of opinions for which the advantage fell in the corresponding interval. The gray part of each bar indicates the proportion of opinions that were liberal opinions, that is, were more common among liberals than among conservatives in the American National Election Studies/General Social Survey data.

Figure 2 shows a histogram of the HVFL argument advantage of the 108 advantaged opinions (27 in the American National Election Studies and 81 in the General Social Survey). The bars of the histogram are color coded in black and gray, where the gray area represents opinions that are more common among liberals than among conservatives in the American National Election Studies and General Social Survey. As predicted, and replicating prior work (Strimling et al., 2019), on the vast majority of moral issues (90 out of 108) the advantaged opinion was also more common among liberals than among conservatives. Moreover, this is likely to be an underestimation of the true connection. Measures of HVFL argument advantage inevitably have some measurement error and exceptions typically occur where the measure of the HVFL argument advantage was <0.1. Our interpretation is that below this threshold we cannot reliably say which opinion truly has the HVFL argument advantage. As the estimated effect of discussion frequency obtains the wrong sign if the HVFL argument has the wrong sign, we therefore use only the issues for which the HVFL argument advantage measure is at least 0.1 in estimations of these effects. This criterion is satisfied by 16 issues in the American National Election Studies and 52 issues in the General Social Survey. However, results are similar if all issues are retained in the analysis.

### Prediction 2: HVFL argument advantage determines opinion change

**Figure 3:**
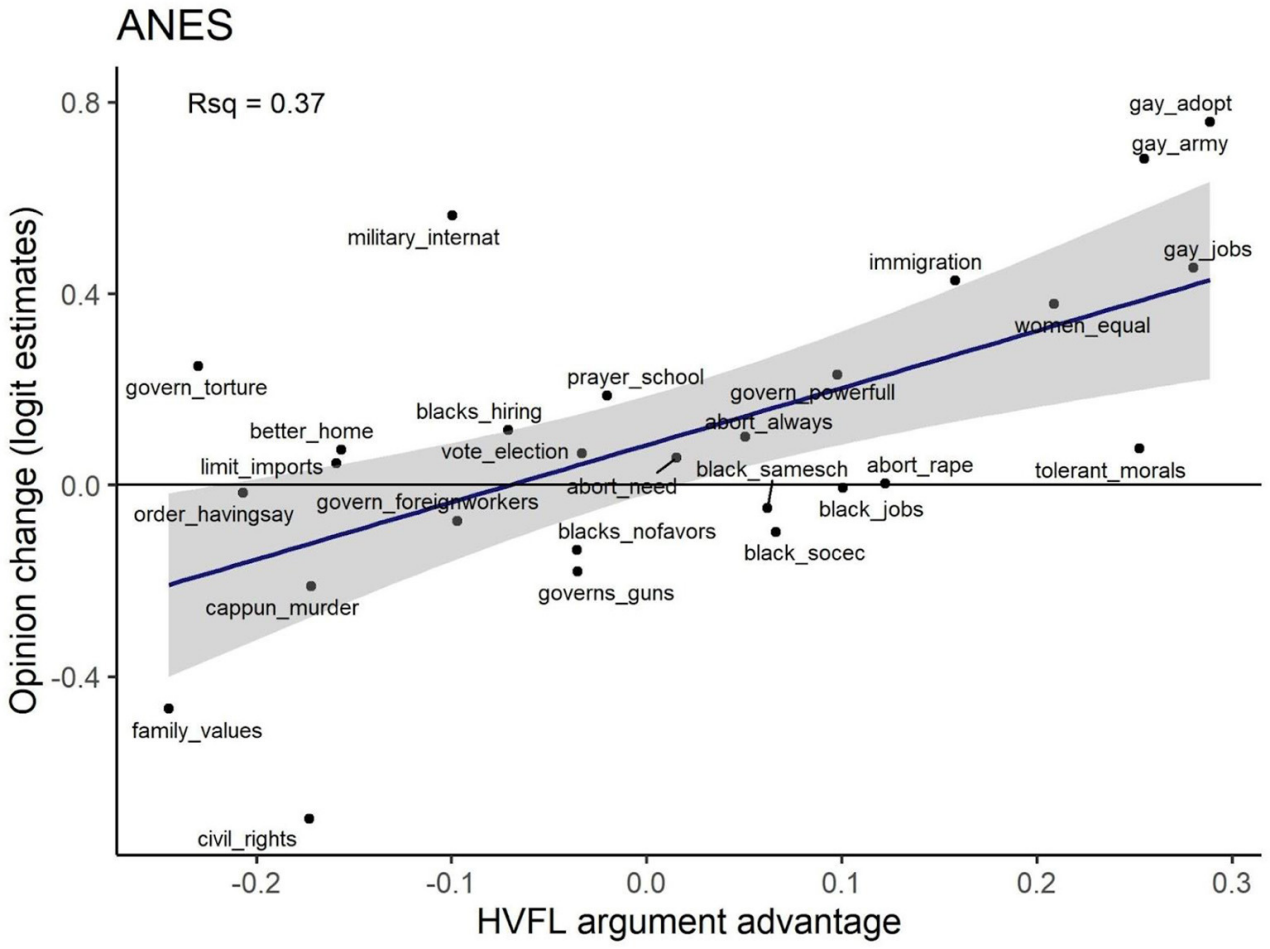
Opinion change is related to the HVFL argument advantage of the opinion. The 27 dots represent items for moral opinions in the American National Election Studies. The labels are our abbreviations of the items (see Supplementary Table 1 for the full items). The y-axis represents estimated opinion change in log odds per 10 years. The x-axis represents the HVFL argument advantage of the opinion stated in the item.

As predicted, and consistent with prior work (Strimling et al., 2019; Eriksson et al., 2022), HVFL argument advantage is strongly associated with estimates of opinion change for the 27 American National Election Studies items, Pearson's *r* = 0.61, 95% CI [0.30, 0.80]. See Figure 3.

### Prediction 3: A systematic discussion frequency effect on liberals' opinions

**Figure 4:**
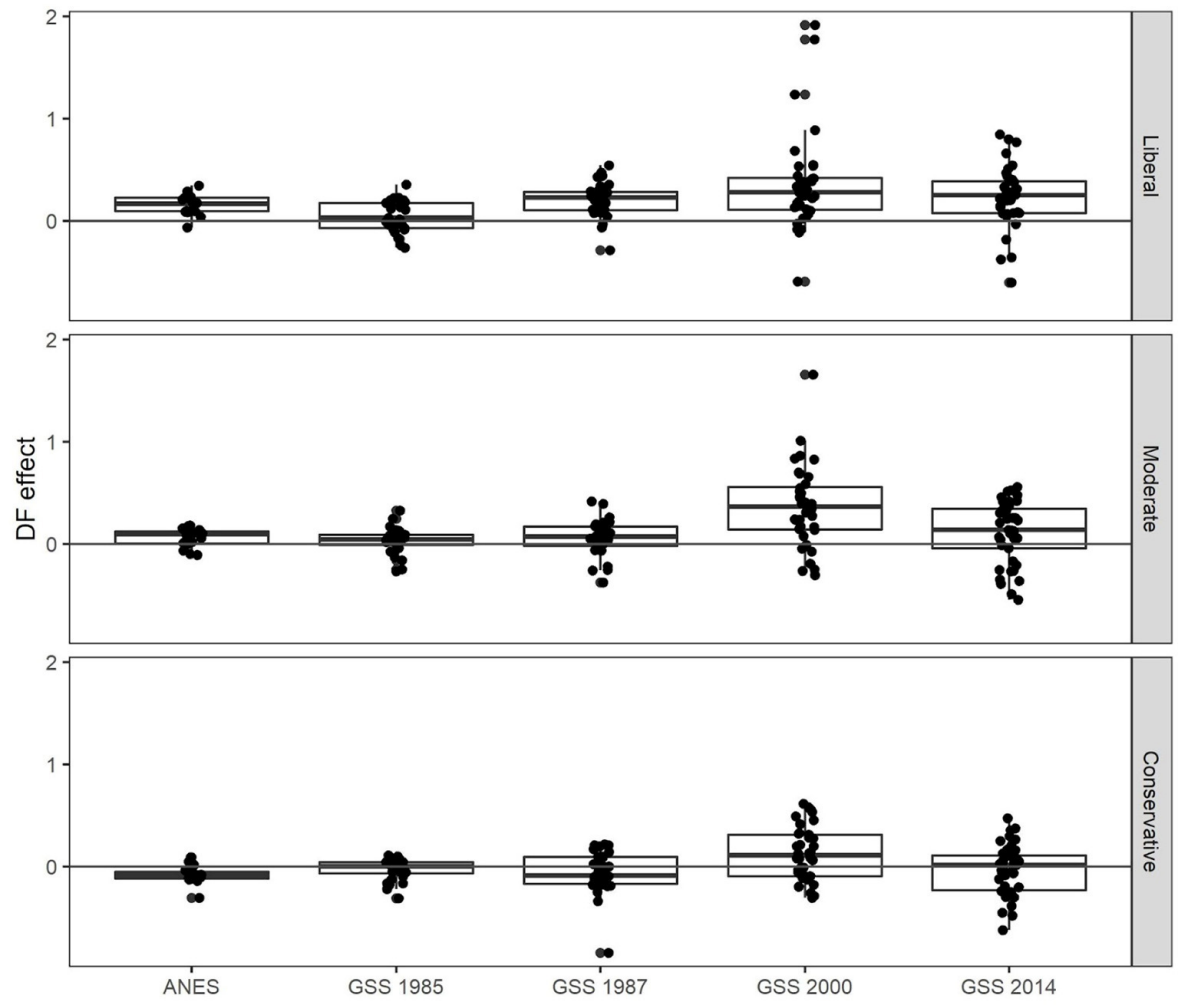
Liberals who discuss more frequently tend to have more liberal opinions, whereas there is no systematic effect of discussion frequency among conservatives. Boxplots of the discussion frequency effect on opinions among liberals (top), moderates (middle), and conservatives (bottom), estimated for 16 moral issues in the American National Election Studies (ANES) and 52 moral issues in the General Social Survey (GSS) using logistic regressions adjusted for education, gender, age and ethnicity. On the vast majority of issues, higher discussion frequency was associated with a greater probability of holding the liberal (HVFL argument advantaged) opinion among liberals, whereas there was no such tendency among conservatives.

When we estimate the discussion frequency effect among liberals separately for each issue in each wave of the General Social Survey, we obtain the results shown in the top panel of Figure 4. Across the American National Election Studies and the General Social Survey, the vast majority of issues exhibit a positive discussion frequency effect on liberals' opinions.

For a statistical test of the frequency effect, we pooled data across all waves and 52 issues in the General Social Survey. As predicted, the average effect of discussion frequency on the probability of holding advantaged opinions among liberals is positive, 0.12, 95% CI [0.05, 0.19]. Similar results were obtained in the American National Election Studies. Across all waves and 16 issues, the average discussion frequency effect among liberals in the American National Election Studies is 0.12, 95% CI [0.08, 0.17]. The full analysis is reported in Supplementary Table 4, Model M1. The interpretation of these results is that an increase in discussion frequency by one standard deviation among liberals is associated with an increase of the odds of holding an opinion with HVFL argument advantage by a factor of exp(0.12) = 1.13.

### Prediction 4: The moderating effect of HVFL advantage on the discussion effect among liberals

**Figure 5:**
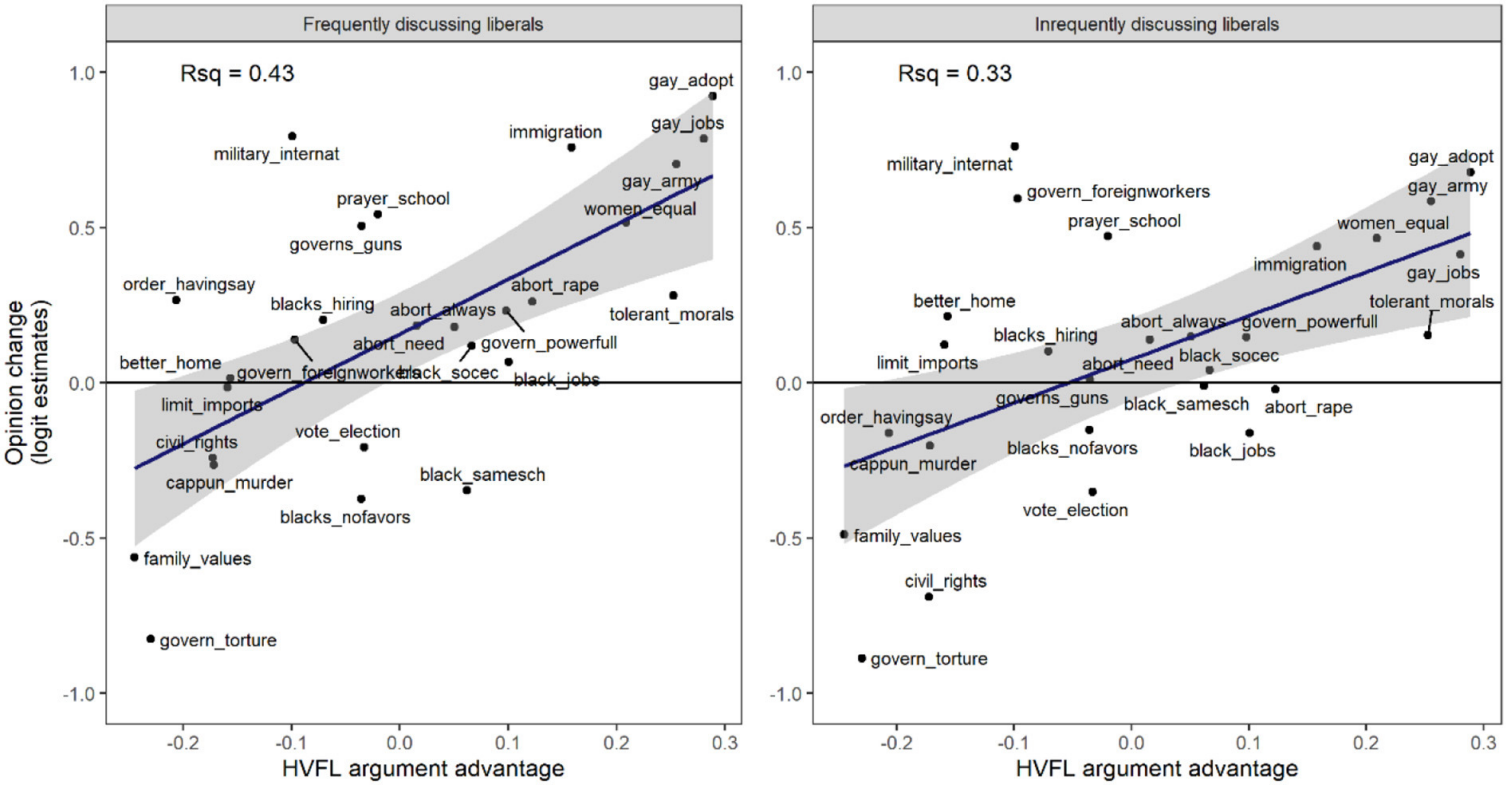
The effect of HVFL argument advantage on opinion change over time in the American National Election Studies is greater among frequently discussing liberals (left) than among infrequently discussing liberals (right). The y-axis represents estimated opinion change in log odds per 10 years. The x-axis represents the HVFL argument advantage of the opinion stated in the item.

As predicted, we also find that the effect of discussion frequency on the opinions among liberals is moderated by the size of the HVFL argument advantage. The interaction was statistically significant in the General Social Survey, 0.10, 95% CI [0.03, 0.16] as well as in the American National Election Studies, 0.08, 95% CI [0.01, 0.14]. The full analysis is reported in Supplementary Table 4, Model M2. The interpretation of the result for the American National Election Studies is that on an issue with a HVFL argument advantage that is 0.1 greater than average, the discussion frequency effect among liberals is exp(0.08) = 1.08 times higher than on an average issue. The moderating effect can also be explored in terms of opinion change. Figure 5 shows the results of estimating opinion change specifically among frequently and infrequently discussing liberals and examining how they relate to the HVFL argument advantage of the opinions. Between the left panel and the right panel there is a difference in slope in the expected direction, 0.36, 95% CI [−0.20, 0.93]. The difference in slope is clear to the eye, but with only 27 datapoints the statistical power is not sufficient for this difference to be statistically significant.

### Prediction 5: Little systematic discussion frequency effect on conservatives' opinions

Finally, we analyzed data on conservatives and moderates instead of liberals, see the middle and bottom panels of Figure 4. As predicted, there is little systematic effect of discussion frequency on the probability of conservatives holding advantaged opinions. The estimated effect was 0.01, 95% CI [−0.05, 0.07] in the General Social Survey data and −0.06 95% CI [−0.10, −0.03] in the American National Election Studies data, see Supplementary Table 5 for full analyses. For moderates, the corresponding results lay in-between the results for liberals and conservatives with an estimated effect of 0.07, 95% CI [0.01, 0.13] in the General Social Survey data and 0.03 95% CI [−0.01, 0.07] in the American National Election Studies data.

## Discussion

In this paper we have considered whether individuals' political discussion frequency has any bearing on their moral opinions. Among liberals we found a systematic effect: Those liberals that discuss more frequently tend to have more liberal opinions on moral issues. This finding holds after controlling for education, gender, age and ethnicity. It is also robust across different data sources using different measures of discussion frequency.

Could it be that discussing has a general effect of making people's opinions more liberal? If so, we should see the same liberalizing effect among conservatives—but we did not. Or could it be that discussing has a general effect of making people's opinions more stereotypical of their ideological group? If so, we should see that higher discussion frequency makes conservatives hold more conservative opinions—but in fact we found little systematic effect of discussion frequency on conservatives' opinions at all. The theoretical challenge is to explain why there is a systematic effect of discussion frequency specifically among liberals.

We have offered a theoretical framework in which people's opinions are formed in a social process in which individual differences in discussion frequency interact with differences between liberals and conservatives in whether or not they mainly accept the HVFL kinds of arguments (harm, violence, fairness, liberty), with a key background variable being the extent to which HVFL arguments favor one opinion over the other on a given moral issue. In a computational model we found that this theory can account for the observed pattern of results for the discussion frequency effects among liberals and conservatives.

A strength of this framework is that opinions on multiple different issues can be studied simultaneously. It only requires that the HVFL argument advantage is measured for the opinions on each issue. Such measures have previously been used to characterize specific opinions as liberal or conservative and to predict opinion trends (Strimling et al., 2019, 2022; Eriksson et al., 2022). Here we replicated these findings. In addition, we found that the size of the HVFL argument advantage predicts how the size of the liberalizing effect of a high discussion frequency varies across different moral issues. These findings provide further evidence of the usefulness of analyzing the kinds of arguments that are used to justify different moral opinions. A crucial property of the HVFL argument advantage measure is that liberals and conservatives agree on it. Thus, it is not the case that people's own opinions are an important determinant of which arguments they see bearing on an issue. This is important when considering the possible directions of causality. The proposed causal direction, from argument advantage to opinion change, is also supported by the finding that argument advantage measures predict future opinion change (Strimling et al., 2022).

To estimate the effect of discussion frequency on opinions we used polling data from the American National Election Studies and the General Social Survey. Due to limited sample sizes, these estimates will have some measurement errors. A more serious limitation is that while our theory speaks about the long-term effects of discussion frequency, we only have data on how often respondents discuss at one point in time. These data will have limited reliability as indicators of how often the respondent has participated in discussions in a longer time perspective. Our estimates of discussion frequency effect will therefore be noisy. However, we see no reason why they would be biased in favor of our theoretical predictions. It is therefore likely that the true effects of discussion frequency on opinions among liberals are even larger and even more systematic.

There are also limitations with respect to the interaction between discussion frequency and the HVFL argument advantage. In addition to the noise in estimates of the discussion frequency effect, there are measurement errors in the HVFL argument advantage measures. Again, we see no reason why the noise would be biased in favor of our theoretical predictions. We therefore expect the estimated interaction to be an underestimation.

Our study is limited in that no data is available on how often specific issues are discussed. It is also possible that discussion frequency is confounded with another variable that affects opinions. Our analysis already controls for a measure of media use, but we cannot exclude other confounders.

## Conclusion

Prior literature has examined how political discussion frequency relates to variables such as political participation, political knowledge, and accuracy of beliefs. Here we have extended the study of political discussion frequency to its effects on the opinions of liberals and conservatives. We found a liberalizing effect on liberals' opinions but little systematic effect on conservatives' opinions. Note that this means that the difference in opinions between liberals and conservatives is larger among those who discuss more frequently than among those who discuss less frequently. Thus, an interpretation of our empirical finding is that more frequent discussions contribute to further polarization of opinions. The moral argument theory of opinion change says that this is the wrong interpretation, however. It says that the discussion frequency effect arises from a social dynamical process that over time makes opinions more liberal both among liberals and conservatives, so that liberals that discuss frequently are just at the forefront of a common trajectory. The common trajectory of the moral opinions of conservatives and liberals has been clearly demonstrated in analyses of General Social Survey data (Strimling et al., 2019).

## Data availability statement

The datasets presented in this study can be found in online repositories. The public opinion data used in the paper are available at the following repositories: https://electionstudies.org/data-center/ (ANES) and https://gss.norc.org/Get-The-Data (GSS). Measures of the HVFL argument advantage together with code that reproduce the analysis are publicly available at https://github.com/irinavrt/mo-discussion-frequency.

## Ethics statement

Ethical review and approval was not required for the study on human participants in accordance with the local legislation and institutional requirements. The patients/participants provided their written informed consent to participate in this study.

## Author contributions

IV collected and collated data and performed the statistical analyses. KE and PS developed the computational model. KE performed the simulations and wrote the manuscript. PS contributed critical revisions. All authors contributed to conception and design of the study and read and approved the submitted version.

## Funding

This work was supported by the Swedish Research Council [2019-02759] and the Knut and Alice Wallenberg Foundation [2017.0257].

## Conflict of interest

The authors declare that the research was conducted in the absence of any commercial or financial relationships that could be construed as a potential conflict of interest.

## Publisher's note

All claims expressed in this article are solely those of the authors and do not necessarily represent those of their affiliated organizations, or those of the publisher, the editors and the reviewers. Any product that may be evaluated in this article, or claim that may be made by its manufacturer, is not guaranteed or endorsed by the publisher.

## Footnotes

^1^In addition to this item, early waves of the American National Election Studies also included an item on how frequently the respondent discusses in general. Although not reported here, this item yields similar patterns of results.
