# Supplementary material for: How does political discussion frequency impact political moral opinions? The moral argument theory of opinion dynamics
Source: Front Psychol. 2022 Sep 2;13:915252. doi: 10.3389/fpsyg.2022.915252 (PMC9479318; doi:10.3389/fpsyg.2022.915252)
Supplement: Supplementary file 1 [file Data_Sheet_1.docx]

**Supplementary material to “How Does Political Discussion Frequency Impact Political Moral Opinions? The Moral Argument Theory of Opinion Dynamics”**

# The computational model

# Text of the questionnaire used to measure the HVFL argument advantage

# Supplementary Table S1. Items for moral opinions in the ANES, with their HVFL argument advantage.

# Supplementary Table S2. Items for moral opinions in the GSS, with their HVFL argument advantage.

# Supplementary Table S3. The set of moral arguments used in the study.

# Supplementary Table S4. Mixed effects logistic regressions of opinions among liberals.

# Supplementary Table S4.A The same as S4 but includes control variables of media consumption.

# Supplementary Table S5. Mixed effects logistic regressions of opinions among conservatives.

# 1. The computational model

***Model assumptions***

The model assumes a population that is sufficiently large for stochastic effects to be ignored. Agents in the population meet at random to discuss an issue on which there are two possible opinions, one of which is “advantaged” in the sense that it has an HVFL argument advantage over the opposite opinion. The aim of the model is to examine how opinions develop among liberal and conservative agents of high and low discussion frequency. These parameters could be viewed as continuous, but for simplicity and illustrative purposes we model them as dichotomous. Agents in the model are therefore defined by their combination of ideological group membership (liberal vs. conservative) and their discussion frequency (hi vs. lo). All four combinations are assumed to include a quarter of the population.

Following Strimling et al. (2019), the HVFL advantage of the advantaged opinion is implemented in terms of switching probabilities for liberal agents. For a liberal agent encountering someone with the opposite opinion, the probability of switching to the advantaged opinion (*b_f_* ) is greater than the probability of switching to the disadvantaged opinion (*b_a_*), and the size of the difference reflects the size of the HVFL argument advantage. For a conservative agent, by contrast, the switching probability is the same in both directions (*b*).

Ingroup bias in discussion partners is defined by the probability that agents discuss with someone from their own ideological group; this bias (*B*_hi_ or *B*_lo_) is assumed to depend only on the agent’s own discussion frequency. Within a given ideological group, the probabilities of discussing with the hi type vs. the lo type are assumed to be proportional to discussion frequencies of each type, denoted by *r*_hi_ and *r*_lo_, respectively, with 0 < *r*_lo_ < *r*_hi_ and scaled so that *r*_hi_ + *r*_lo_ = 1.

***Deriving model dynamics***

The model dynamics proceed in discrete time steps. To examine how opinions change in the stratified population, we track the proportions holding the advantaged opinion at time *t* among hi liberals, lo liberals, hi conservatives, and lo conservatives: *ℓ*_hi_(*t*), *ℓ*_lo_(*t*), *c*_hi_(*t*), *c*_lo_(*t*). Let Δ *ℓ*_hi_ = *ℓ*_hi_ (*t*+1) - *ℓ*_hi_ (*t*) denote the rate of change of *ℓ*_hi_. To avoid cumbersome notation, we will otherwise not write out the dependency on *t.* An agent discussing with a liberal will encounter the advantaged opinion with probability *ℓ* = *r*_hi_ *ℓ*_hi_ + *r*_lo_ *ℓ*_lo_, while an agent discussing with a conservative will encounter the advantaged opinion with probability *c* = *r*_hi_ *c*_hi_ + *r*_lo_ *c*_lo_.

The rate of change of *ℓ*_hi_ can be calculated as the discussion frequency of hi agents times the difference in probability between the event that a hi liberal agent who holds the disadvantaged opinion switches upon meeting an agent who holds the advantaged opinion and the event that a hi liberal agent who holds the advantaged opinion switches after meeting an agent who holds the disadvantaged opinion:

(1) Δ *ℓ*_hi_ = *r*_hi_[(1-*ℓ*_hi_ )(*B*_hi_ *ℓ*+(1-*B*_hi_)*c*)*b_f_* - *ℓ*_hi_ (*B*_hi_ (1-*ℓ*)+(1-*B*_hi_)(1-*c*))*b_a_*].

By changing every hi to lo in equation (1) we obtain a description of the rate of opinion change among lo liberals:

(2) Δ *ℓ*_lo_ = *r*_lo_[(1-*ℓ*_lo_ )(*B*_lo_ *ℓ*+(1-*B*_lo_)*c*)*b_f_* - *ℓ*_lo_ (*B*_lo_ (1-*ℓ*)+(1-*B*_lo_)(1-*c*))*b_a_*].

To obtain the formulas for conservatives, change every *ℓ* to a *c* and vice versa, and replace both *b_f_* and *b_a_* by *b.* After simplification, the formula for opinion change among hi conservatives becomes

(3) Δ *c*_hi_ = *r*_hi_*b*[*B*_hi_(*c*-*c*_hi_) + (1-*B*_hi_)(*ℓ* - *c*_hi_)]

and the formula for lo conservatives becomes

(4) Δ *c*_lo_ = *r*_lo_*b*[*B*_lo_(*c*-*c*_lo_) + (1-*B*_lo_)(*ℓ* - *c*_lo_)].

***Model outcomes***

Equipped with these dynamical equations, we can set an initial distribution of opinions and examine the evolution over time of an effect of discussion frequency on the opinions among liberals (*ℓ*_hi_  - *ℓ*_lo_) as well as the corresponding effect among conservatives (*c*_hi_  - *c*_lo_). As our initial state we choose a distribution where the proportion holding the advantaged opinion is just 1% within the whole population, independent of ideological group and discussion frequency: *ℓ*_hi_(0) = *ℓ*_lo_(0) = *c*_hi_(0) = *c*_lo_(0) = 0.01. Note that the property *c*_hi_ = *c*_lo_ = *c* in the first time step means that equations (3) and (4) reduce to

(5) Δ *c*_hi_ = *r*_hi_*b*(1-*B*_hi_)(*ℓ* - *c*) and Δ *c*_lo_ = *r*_lo_*b*(1-*B*_lo_)(*ℓ* - *c*).

If we choose the parameter values *r*_hi_, *B*_hi_, *r*_lo_, and *B*_lo_ so that *r*_hi_(1-*B*_hi_) = *r*_lo_(1-*B*_lo_), then equation (5) yields Δ *c*_hi_ = Δ *c*_lo_, that is, *c*_hi_ and *c*_lo_ will remain equal. Hence the effect of discussion frequency on the opinions among conservatives will be constant at zero. This case is illustrated in Figure S1 for two different sizes of the HVFL argument advantage: a small advantage in Model A, a big advantage in Model B. Blue lines show how the frequency effect on the opinions among liberals evolves in a simulation of the dynamical system as the advantaged opinion grows in popularity from 1% to 99% of the whole population. Red lines show the constant zero effect of discussion frequency on the opinions among conservatives. There are two take-home messages from Figure S1: First, although the frequency effect among conservatives is zero, the frequency effect among liberals is positive. Second, the frequency effect among liberals grows with the size of the HVFL argument advantage.


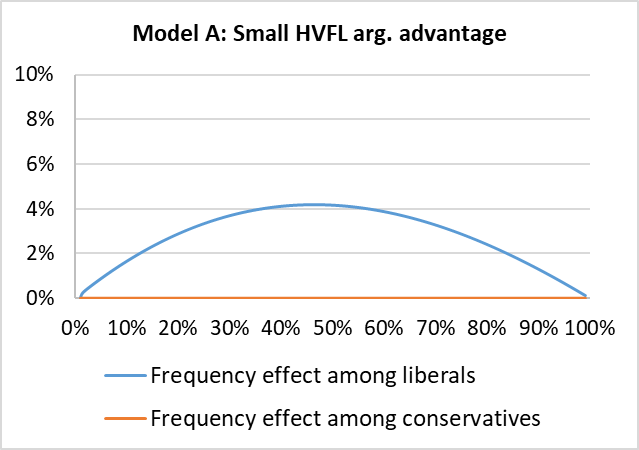

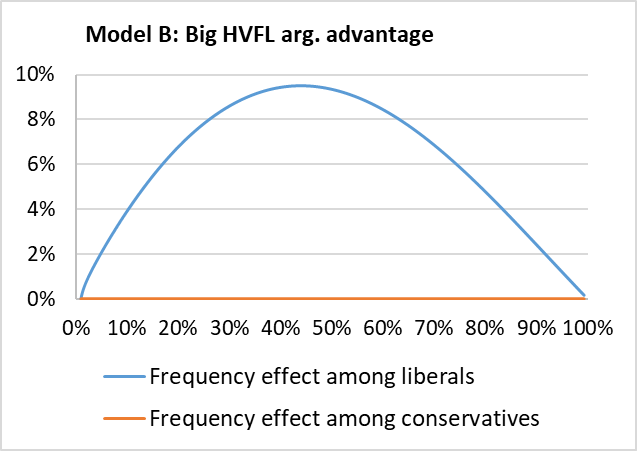


**Figure S1**. **The frequency effect on the opinions of liberals is positive and grows with the size of the HVFL argument advantage.** Simulations of the evolution of the effect of discussion frequency on the probability of holding the opinion with HVFL argument advantage among liberals (blue line, representing *ℓ*_hi_  - *ℓ*_lo_) and conservatives (red line, representing *c*_hi_  - *c*_lo_) as the proportion of the advantaged opinion in the whole population grows from 1% to 99% (x-axis). Both models use discussion frequencies *r*_hi_ = ⅔ and *r*_lo_ = ⅓, and ideological ingroup biases *B*_hi_ = 0.75 and *B*_lo_ = 0.5 (i.e., no bias in the lo type). Model A is defined by a “small” HVFL argument advantage (*b_a_* = 0.008 and *b_f_* = *b* = 0.01) compared to a “big” HVFL argument advantage in model B (*b_a_* = 0.006 and *b_f_* = *b* = 0.01).

The assumption that *r*_hi_(1-*B*_hi_) = *r*_lo_(1-*B*_lo_) was only made for illustrative purposes. If we fix the discussion frequencies of the hi and low types (*r*_hi_ and *r*_lo_), the remaining question is what happens when we vary the ratio of the ideological ingroup bias of hi types and lo types (*B*_hi_ : *B*_lo_). This is illustrated in Figure S2, which shows two extreme cases: equal bias in both two types (Model C) and maximal bias in the hi type while the lo type is unbiased (Model D). In the former case, there is a positive frequency effect also among conservatives but not as large as the effect among liberals (except when the advantaged opinion almost saturates the population). In the latter case, there is a negative frequency effect among conservatives but not as large as the positive effect among liberals. Thus, for a wide range of possible parameter values, the model predicts a stronger frequency effect among liberals than among conservatives. Consequently, the frequency effect in the whole population (i.e., the average of the effects among liberals and among conservatives) is positive.


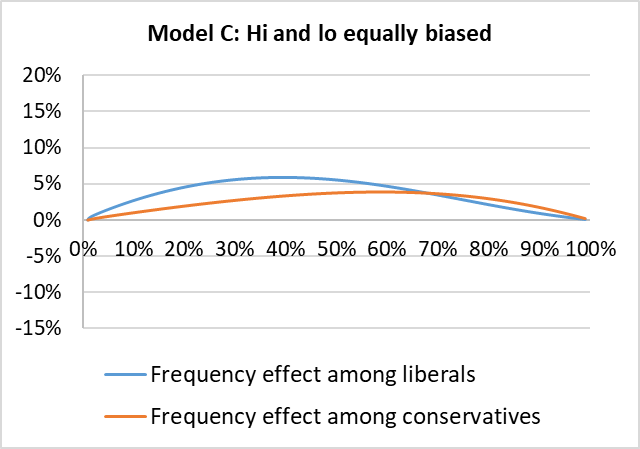

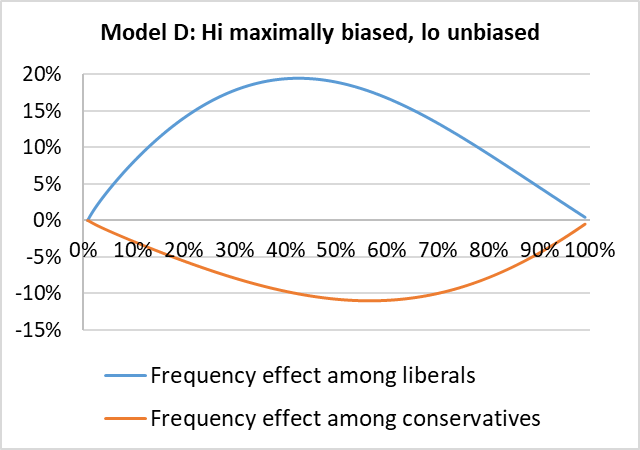


**Figure S2**. **The frequency effect remains smaller among conservatives than among liberals as the ingroup bias ratio varies.** Simulations of the evolution of the effect of discussion frequency on the probability of holding the opinion with HVFL argument advantage among liberals (blue line, representing *ℓ*_hi_  - *ℓ*_lo_) and conservatives (red line, representing *c*_hi_  - *c*_lo_) as the proportion of the advantaged opinion in the whole population grows from 1% to 99% (x-axis). Both models use discussion frequencies *r*_hi_ = ⅔ and *r*_lo_ = ⅓, and a “big” HVFL argument advantage (*b_a_* = 0.006 and *b_f_* = *b* = 0.01). Model C is defined by hi and lo types having equal ideological ingroup bias, *B*_hi_ = *B*_lo_ = 0.75. Model D is defined by the hi type having maximal bias, *B*_hi_ = 1, and the lo type having no bias, *B*_lo_ = 0.5.

**2. Text of the questionnaire used to measure the HVFL argument advantage.** Two of the 27 items are shown in this excerpt.

In the following section, you will be asked to provide your opinion on 9 issues and choose very general arguments from a list that could support your (and the opposite) position on these issues. At the end there is a code that you can enter into mturk to receive your payment.

By submitting this form you consent to your answers being used, anonymously, as data for our study.

Thank you!

Please fill out your answer to this question or statement:

**Some people say that the government in Washington should see to it that white and black children go to the same schools. Others claim this is not the government's business. What about you: should the government see to it that white and black children go to the same schools?**

- Yes
- No

*Some people say that the government in Washington should see to it that white and black children go to the same schools. Others claim this is not the government's business. What about you: should the government see to it that white and black children go to the same schools?*

**Now consider why you chose that answer. Which of the following arguments apply? Please tick all that apply. Yes, because ...**

- then someone cares for someone weak or vulnerable
- otherwise some people are treated differently from others
- then everyone is free to do as they wanted
- then someone conforms to the traditions of society
- otherwise someone violates standards of purity and decency
- then someone's action shows love for his or her country
- otherwise someone is killed
- of some other reason (specify)

*Some people say that the government in Washington should see to it that white and black children go to the same schools. Others claim this is not the government's business. What about you: should the government see to it that white and black children go to the same schools?*

**Now we would also like you to consider which of the arguments you would expect from someone who had answered No to the question. They would answer No because ...**

- then some people are treated differently from others
- then someone does not conform to the traditions of society
- then someone's action does not show love for his or her country
- then someone does not care for someone weak or vulnerable
- then someone is killed
- then someone violates standards of purity and decency
- then everyone is not free to do as they wanted
- of some other reason (specify)

Please fill out your answer to this question or statement:

**We should be more tolerant of people who choose to live according to their own moral standards, even if they are very different from our own.**

- Yes
- No

*We should be more tolerant of people who choose to live according to their own moral standards, even if they are very different from our own.*

**Now consider why you chose that answer. Which of the following arguments apply? Please tick all that apply. No, because ...**

- then someone suffers emotionally
- then someone does something to betray his or her group
- then someone acts in a way that God would not approve of
- then someone acts unfairly
- then someone creates disruption to the order in our country
- then violence is used
- then someone's freedom of choice is restricted
- of some other reason (specify)

*We should be more tolerant of people who choose to live according to their own moral standards, even if they are very different from our own.*

**Now we would also like you to consider which of the arguments you would expect from someone who had answered Yes to the question. They would answer Yes because ...**

- otherwise someone's freedom of choice is restricted
- otherwise someone creates disruption to the order in our country
- otherwise someone acts unfairly
- otherwise violence is used
- otherwise someone suffers emotionally
- then someone acts in a way that God would approve of
- otherwise someone does something to betray his or her group
- of some other reason (specify)

# Supplementary Table S1. Items for moral opinions in the ANES, with their HVFL argument advantage.

| **ANES item** | **HVFL** |
| --- | --- |
| Do you think gay or lesbian couples, in other words, homosexual couples, should be legally permitted to adopt children? | 0.29 |
| Do you favor laws to protect homosexuals (gays and lesbians) against job discrimination? | 0.28 |
| Do you think homosexuals should be allowed to serve in the United States Armed Forces? | 0.26 |
| We should be more tolerant of people who choose to live according to their own moral standards, even if they are very different from our own. | 0.25 |
| This country would have many fewer problems if there were more emphasis on traditional family ties. | -0.24 |
| Do you favor the U.S. government torturing people, who are suspected of being terrorists, to try to get information? | -0.23 |
| Recently there has been a lot of talk about women’s rights. Some people feel that women should have an equal role with men in running business, industry and government. Others feel that a woman’s place is in the home. What about you: should women have an equal role in running business, industry and government? | 0.21 |
| Do you think that maintaining order in the nation is more important than giving people more say in important government decisions? | -0.21 |
| Some say that the civil rights people have been trying to push too fast. Others feel they haven’t pushed fast enough. How about you: Do you think that civil rights leaders are trying to push too fast? | -0.17 |
| Do you favor the death penalty for persons convicted of murder? | -0.17 |
| Some people have suggested placing new limits on foreign imports in order to protect American jobs. Others say that such limits would raise consumer prices and hurt American exports. What about you, do you favor placing new limits on imports? | -0.16 |
| Do you think the number of immigrants from foreign countries who are permitted to come to the United States to live should be increased? | 0.16 |
| This country would be better off if we just stayed home and did not concern ourselves with problems in other parts of the world. | -0.16 |
| There has been some discussion about abortion during recent years. Do you think that the law should permit abortion in case of rape, incest, or when the woman’s life is in danger? | 0.12 |
| Some people feel that if black people are not getting fair treatment in jobs, the government in Washington ought to see to it that they do. Others feel that this is not the federal government’s business. Should the government in Washington see to it that black people get fair treatment in jobs? | 0.10 |
| Do you think the United States should be willing to use military force to solve international problems in the future? | -0.10 |
| Some people are afraid the government in Washington is getting too powerful for the good of the country and the individual person. Others feel that the government in Washington is not getting too strong. What is your feeling? Do you think the government is too powerful? | 0.10 |
| Recently, some big American companies have been hiring workers in foreign countries to replace workers in the U.S. Do you think the federal government should discourage companies from doing this? | -0.10 |
| Because of past discrimination blacks should be given preference in hiring and promotion. | -0.07 |
| Some people feel that the government in Washington should make every effort to improve the social and economic position of blacks. Others feel that the government should not make any special effort to help blacks because they should help themselves. What is your opinion: should the government in Washington make every effort to improve the social and economic position of blacks, rather than letting them help themselves? | 0.07 |
| Some people say that the government in Washington should see to it that white and black children go to the same schools. Others claim this is not the government’s business. What about you: should the government see to it that white and black children go to the same schools? | 0.06 |
| There has been some discussion about abortion during recent years. Do you think that by law, a woman should always be able to obtain an abortion as a matter of personal choice? | 0.05 |
| Irish, Italians, Jewish and many other minorities overcame prejudice and worked their way up. Blacks should do the same without any special favors. | -0.04 |
| Do you think the federal government should make it more difficult for people to buy a gun than it is now? | -0.04 |
| If a person doesn’t care how an election comes out they shouldn’t vote in it. | -0.03 |
| By law, prayer should not be allowed in public schools. | -0.02 |
| There has been some discussion about abortion during recent years. Do you think that the law should permit abortion after the need for the abortion has been clearly established? | 0.02 |

#

# Supplementary Table S2. Items for moral opinions in the GSS, with their HVFL argument advantage.

| GSS item | HVFL |
| --- | --- |
| Homosexual couples should have the right to marry one another. | 0.41 |
| Women should take care of running their homes and leave running the country up to men. | -0.37 |
| Do you think there should be laws against marriages between African-Americans and whites? | -0.37 |
| Would you be in favor of having a close relative or family member marry a black person? | 0.36 |
| Consider a man who admits that he is a homosexual. Should such a person be allowed to teach in a college or university? | 0.35 |
| Would you approve of a man punching a stranger who was in a protest march showing opposition to the other man's views? | -0.35 |
| Consider a man who admits that he is a homosexual. If some people in your community suggested that a book he wrote in favor of homosexuality should be taken out of your public library -- would you favor removing this book? | -0.33 |
| Would you be in favor of having a close relative or family member marry an Asian American person? | 0.32 |
| It is much better for everyone involved if the man is the achiever outside the home and the woman takes care of the home and family. | -0.32 |
| Consider a man who admits he is a Communist. Suppose he is teaching in a college. Should he be fired? | -0.30 |
| Would you approve of a policeman striking a citizen who had said vulgar and obscene things to the policeman? | -0.30 |
| Would you approve of a policeman striking a citizen who was being questioned as a suspect in a murder case? | -0.29 |
| Would you be in favor of having a close relative or family member marry a Hispanic American person? | 0.29 |
| What about sexual relations between two adults of the same sex--do you think it is always wrong? | -0.29 |
| Consider a man who admits that he is a homosexual? Suppose this admitted homosexual wanted to make a speech in your community. Should he be allowed to speak? | 0.29 |
| Do you agree that methods of birth control should be available to teenagers between the ages of 14 and 16 if their parents do not approve? | 0.28 |
| It is more important for a wife to help her husband's career than to have one herself. | -0.28 |
| Do you approve of a married woman earning money in business or industry if she has a husband capable of supporting her? | 0.27 |
| White people have a right to keep African-Americans out of their neighborhoods if they want to and African-Americans should respect that right. | -0.27 |
| African-Americans shouldn't push themselves where they're not wanted. | -0.26 |
| Consider a man who admits he is a Communist. Suppose he wrote a book which is in your public library. Somebody in your community suggests that the book should be removed from the library. Would you favor removing it? | -0.26 |
| Do you agree that it is sometimes necessary to discipline a child with a good hard spanking? | -0.25 |
| If you and your friends belonged to a social club that would not let African-Americans join -- would you try to change the rules so that African-Americans could join? | 0.25 |
| There's been a lot of discussion about the way morals and attitudes about sex are changing in this country. If a man and woman have sex relations before marriage -- do you think it is wrong? | -0.24 |
| In general some people think that it is the responsibility of the government in Washington to see to it that people have help in paying for doctors and hospital bills. Others think that these matters are not the responsibility of the federal government and that people should take care of these things themselves. Do you think that it is the responsibility of the government? | 0.24 |
| There are always some people whose ideas are considered bad or dangerous by other people. For instance: somebody who is against all churches and religion -- should such a person be allowed to teach in a college or university? | 0.24 |
| Consider a person who advocates doing away with elections and letting the military run the country. Suppose he wrote a book advocating doing away with elections and letting the military run the country. Somebody in your community suggests that the book be removed from the public library. Would you favor removing it? | -0.23 |
| Do you think the use of marijuana should be made legal? | 0.23 |
| Consider a man who admits he is a Communist. Suppose this admitted Communist wanted to make a speech in your community. Should he be allowed to speak? | 0.23 |
| There are always some people whose ideas are considered bad or dangerous by other people. For instance somebody who is against all churches and religion. If some people in your community suggested that a book he wrote against churches and religion should be taken out of your public library -- would you favor removing this book? | -0.23 |
| Would you approve of a policeman striking a citizen who was attempting to escape from custody? | -0.23 |
| Would you be in favor of having a close relative or family member marry a white person? | 0.21 |
| Consider somebody who is against all churches and religion. If such a person wanted to make a speech in your (city/town/community) against churches and religion -- should he be allowed to speak? | 0.21 |
| Some people think that the government in Washington should do everything possible to improve the standard of living of all poor Americans. Other people think it is not the government's responsibility and that each person should take care of himself. Do you think that the government should do everything possible to improve the standard of living of all poor Americans? | 0.18 |
| When a person has a disease that cannot be cured -- do you think doctors should be allowed by law to end the patient's life by some painless means if the patient and his family request it? | 0.17 |
| Consider a person who advocates doing away with elections and letting the military run the country. If such a person wanted to make a speech in your community -- should he be allowed to speak? | 0.17 |
| Consider people who are in their early teens -- say 14 to 16 years old. In that case: do you think sex relations before marriage are wrong? | -0.17 |
| Consider your feelings about pornography laws. Do you think it should be illegal? | -0.16 |
| Would you approve of a man punching a stranger who was drunk and bumped into the man and his wife on the street? | -0.16 |
| Consider a person who advocates doing away with elections and letting the military run the country. Should such a person be allowed to teach in a college or university? | 0.16 |
| Do you think it should be possible for a pregnant woman to obtain a legal abortion if the woman's own health is seriously endangered by the pregnancy? | 0.16 |
| Now consider a Muslim clergyman who preaches hatred of the United States. If such a person wanted to make a speech in your community preaching hatred of the United States - should he be allowed to speak? | 0.15 |
| Do you think it should be possible for a pregnant woman to obtain a legal abortion if she became pregnant as a result of rape? | 0.14 |
| Do you favor the death penalty for persons convicted of murder? | -0.14 |
| Now consider a Muslim clergyman who preaches hatred of the United States. Should such a person be allowed to teach in a college or university? | 0.14 |
| Some people think that African-Americans have been discriminated against for so long that the government has a special obligation to help improve their living standards. Others believe that the government should not be giving special treatment to African-Americans. Do you think that the government should help improve the living standards of African-Americans? | 0.13 |
| Are there any situations you can imagine in which you would approve of a policeman striking an adult male citizen? | -0.12 |
| Suppose there is a community-wide vote on the general housing issue. There are two possible laws to vote on (OWNER DECIDES and CAN'T DISCRIMINATE). Would you vote for owner decides? | -0.11 |
| Do you think a person has the right to end his or her own life if this person has an incurable disease? | 0.11 |
| Would you be for sex education in the public schools? | 0.11 |
| Everything considered: would you say that -- in general -- you approve of wiretapping? | -0.11 |
| Would you approve of a man punching a stranger who was beating up a woman and the man saw it? | 0.11 |
| In general - would you say that people should obey the law without exception rather than there are exceptional occasions on which people should follow their consciences even if it means breaking the law? | -0.10 |
| Do you think a person has the right to end his or her own life if this person is tired of living and ready to die? | 0.10 |
| Do you think it should be possible for a pregnant woman to obtain a legal abortion if she is not married and does not want to marry the man? | 0.08 |
| Government should let ordinary people decide for themselves how to protect the environment - even if it means they don't always dothe right thing - rather than passing laws to make ordinary people protect the environment as it interferes with people's right to make their own decisions. | 0.08 |
| Would you yourself have any objection to sending your children to a school where a few of the children are African-Americans? | -0.07 |
| What is your opinion about a married person having sexual relations with someone other than the marriage partner--is it wrong? | -0.07 |
| Even if it brings no immediate benefits - scientific research that advances the frontiers of knowledge is necessary and should be supported by the federal government. | 0.07 |
| Would you approve of a policeman striking a citizen who was attacking the policeman with his fists? | -0.06 |
| Do you think it should be possible for a pregnant woman to obtain a legal abortion if the family has a very low income and cannot afford any more children? | 0.06 |
| Would you favor a law which would require a person to obtain a police permit before he or she could buy a gun? | -0.05 |
| Consider a person who believes that Blacks are genetically inferior. If some people in your community suggested that a book he wrote which said Blacks are inferior should be taken out of your public library -- would you favor removing this book? | -0.05 |
| In general: do you favor the busing of African-American and white school children from one school district to another? | 0.05 |
| Are there any situations that you can imagine in which you would approve of a man punching an adult male stranger? | -0.05 |
| Do you think it should be possible for a pregnant woman to obtain a legal abortion if she is married and does not want any more children? | 0.05 |
| Consider a person who believes that Blacks are genetically inferior. Should such a person be allowed to teach in a college or university? | 0.05 |
| Would you yourself have any objection to sending your children to a school where more than half of the children are African-Americans? | -0.05 |
| Would you approve of a man punching a stranger who had broken into the man's house? | -0.04 |
| Do you think a person has the right to end his or her own life if this person has dishonored his or her family? | -0.04 |
| Some people say that because of past discrimination - blacks should be given preference in hiring and promotion. Others say that such preference in hiring and promotion of blacks is wrong because it discriminates against whites. What about your opinion - are you for preferential hiring and promotion of blacks? | -0.04 |
| Do you think it should be possible for a pregnant woman to obtain a legal abortion if there is a strong chance of serious defect in the baby? | 0.03 |
| Irish and Italians and Jewish and many other minorities overcame prejudice and worked their way up. Blacks should do the same without special favors. | -0.02 |
| Would you approve of a man punching a stranger who had hit the man's child after the child accidentally damaged the stranger's car? | -0.02 |
| Government should let businesses decide for themselves how to protect the environment - even if it means they don't always do the right thing - rather than passing laws to make businesses protect the environment as it interferes with business' right to make their own decisions. | 0.02 |
| Would you yourself have any objection to sending your children to a school where half of the children are African-Americans? | -0.02 |
| Consider a person who believes that Blacks are genetically inferior. If such a person wanted to make a speech in your community claiming that Blacks are inferior -- should he be allowed to speak? | 0.01 |
| Some people say that because of past discrimination women should be given preference in hiring and promotion. Others say that such preference in hiring and promotion of women is wrong because it discriminates against men. What about your opinion - are you for preferential hiring and promotion of women? | -0.01 |
| Because of past discrimination employers should make special efforts to hire and promote qualified women. | -0.01 |
| Do you think it should be possible for a pregnant woman to obtain a legal abortion if the woman wants it for any reason? | 0.00 |
| Do you think a person has the right to end his or her own life if this person has gone bankrupt? | 0.00 |

# Supplementary Table S3. The set of moral arguments used in the study.

| Kind | Arguments |
| --- | --- |
| Harm | Someone suffers emotionally |
|  | Someone cares for someone weak or vulnerable |
|  | Someone is cruel |
| Fairness | Some people are treated differently from others |
|  | Someone acts unfairly |
|  | Someone is denied his or her rights |
| Loyalty | Someone's action shows love for his or her country |
|  | Someone does something to betray his or her group |
|  | Someone shows a lack of loyalty |
| Authority | Someone shows a lack of respect for authority |
|  | Someone conforms to the traditions of society |
|  | Someone creates disruption to the order in our country |
| Purity | Someone violates standards of purity and decency |
|  | Someone does something disgusting |
|  | Someone acts in a way that God would approve of |
| Liberty | Everyone is free to do as they wanted |
|  | Someone's freedom of choice is restricted |
|  | Everyone is free to decide what group norms or traditions they want to follow |
| Violence | Violence is used |
|  | Someone is killed |
|  | Someone is physically harmed |

# Supplementary Table S4. Mixed effects logistic regressions of opinions among liberals.

|  | ANES | | | GSS | | |
| --- | --- | --- | --- | --- | --- | --- |
|  | M0 | M1 | M2 | M0 | M1 | M2 |
| (Intercept) | 0.89 [0.18, 1.60] | 0.86 [0.14, 1.57] | 0.84 [0.15, 1.52] | 1.19 [0.77, 1.60] | 1.19 [0.77, 1.60] | 1.17 [0.77, 1.56] |
| Age | -0.09 [-0.13, -0.05] | -0.11 [-0.15, -0.07] | -0.11 [-0.15, -0.07] | -0.32 [-0.38, -0.27] | -0.34 [-0.39, -0.29] | -0.34 [-0.39, -0.29] |
| Education | 0.47 [0.43, 0.51] | 0.44 [0.40, 0.47] | 0.44 [0.40, 0.47] | 0.70 [0.65, 0.76] | 0.68 [0.63, 0.74] | 0.68 [0.63, 0.74] |
| Female | 0.14 [0.07, 0.22] | 0.15 [0.07, 0.22] | 0.15 [0.07, 0.22] | 0.11 [0.00, 0.22] | 0.11 [0.00, 0.22] | 0.11 [0.00, 0.22] |
| Black | -0.24 [-0.35, -0.13] | -0.22 [-0.33, -0.11] | -0.22 [-0.33, -0.11] | -0.18 [-0.33, -0.03] | -0.18 [-0.33, -0.03] | -0.18 [-0.33, -0.03] |
| Other | -0.22 [-0.33, -0.11] | -0.20 [-0.31, -0.09] | -0.20 [-0.31, -0.09] | -0.42 [-0.64, -0.21] | -0.43 [-0.64, -0.21] | -0.43 [-0.64, -0.21] |
| DF |  | 0.18 [0.13, 0.23] | 0.07 [−0.07, 0.20] |  | 0.12 [0.05, 0.20] | 0.12 [0.05, 0.19] |
| HVFL |  |  | 0.72 [-0.44, 1.87] |  |  | 0.58 [0.09, 1.06] |
| DF × HVFL |  |  | 0.06 [−0.01, 0.13] |  |  | 0.10 [0.03, 0.17] |
| σ_individual_ | 0.74 | 0.73 | 0.73 | 0.90 | 0.89 | 0.90 |
| σ_issue-year_ | 0.49 | 0.47 | 0.47 | 0.52 | 0.54 | 0.54 |
| σ_issue_ | 1.44 | 1.44 | 1.38 | 1.43 | 1.43 | 1.35 |
| σ_DFissue-slope_ |  | 0.05 | 0.04 |  | 0.16 | 0.14 |
| N_issue_ | 16 | 16 | 16 | 52 | 52 | 52 |
| N_issue-year_ | 120 | 120 | 120 | 137 | 137 | 137 |
| N_individual_ | 4976 | 4976 | 4976 | 979 | 979 | 979 |
| N_obs_ | 44453 | 44453 | 44453 | 31593 | 31593 | 31593 |
| AIC | 35606.8 | 35527.3 | 35526.7 | 26089.1 | 26039.8 | 26034.6 |
| BIC | 35685.1 | 35631.7 | 35648.5 | 26164.3 | 26140.1 | 26151.6 |

*Note*. Model M0 is a null model of the probability among liberals to hold a moral opinion with HVFL advantage, including only controls. Model M1 estimates the effect of discussion frequency on this probability. Model M2 estimates how this effect is moderated by the size of the HVFL advantage of the opinion.

# Supplementary Table S4.A The same as S4 but includes control variables of media consumption.

|  | ANES | | | GSS | | |
| --- | --- | --- | --- | --- | --- | --- |
|  | M0 | M1 | M2 | M0 | M1 | M2 |
| (Intercept) | 0.70 [-0.02, 1.43] | 0.68 [-0.04, 1.41] | 0.67 [-0.04, 1.38] | 1.19 [0.78, 1.60] | 1.18 [0.77, 1.59] | 1.16 [0.77, 1.56] |
| Age | -0.12 [-0.18, -0.07] | -0.13 [-0.18, -0.08] | -0.13 [-0.18, -0.08] | -0.34 [-0.40, -0.28] | -0.36 [-0.42, -0.30] | -0.36 [-0.42, -0.30] |
| Education | 0.49 [0.44, 0.54] | 0.47 [0.42, 0.51] | 0.47 [0.42, 0.51] | 0.68 [0.62, 0.74] | 0.66 [0.60, 0.72] | 0.66 [0.60, 0.72] |
| Female | 0.19 [0.09, 0.29] | 0.18 [0.09, 0.28] | 0.18 [0.09, 0.28] | 0.13 [0.01, 0.24] | 0.13 [0.02, 0.25] | 0.13 [0.02, 0.24] |
| Black | -0.12 [-0.27, 0.03] | -0.10 [-0.25, 0.04] | -0.11 [-0.25, 0.04] | -0.22 [-0.38, -0.07] | -0.22 [-0.38, -0.06] | -0.22 [-0.38, -0.06] |
| Other | -0.12 [-0.28, 0.03] | -0.10 [-0.26, 0.05] | -0.10 [-0.26, 0.05] | -0.53 [-0.77, -0.30] | -0.52 [-0.75, -0.28] | -0.52 [-0.76, -0.29] |
| News | 0.00 [-0.05, 0.05] | -0.02 [-0.07, 0.03] | -0.02 [-0.07, 0.03] | 0.04 [-0.02, 0.10] | 0.02 [-0.04, 0.08] | 0.02 [-0.04, 0.08] |
| DF |  | 0.12 [0.08, 0.17] | 0.12 [0.07, 0.17] |  | 0.11 [0.03, 0.18] | 0.11 [0.04, 0.18] |
| HVFL |  |  | 0.53 [-0.67, 1.72] |  |  | 0.56 [0.08, 1.03] |
| DF × HVFL |  |  | 0.08 [0.01, 0.14] |  |  | 0.09 [0.02, 0.16] |
| σ_individual_ | 0.70 | 0.69 | 0.69 | 0.90 | 0.89 | 0.89 |
| σ_issue-year_ | 1.46 | 1.46 | 1.43 | 1.41 | 1.41 | 1.34 |
| σ_issue_ | 0.36 | 0.35 | 0.35 | 0.52 | 0.52 | 0.52 |
| σ_DFissue-slope_ |  | - | - |  | 0.14 | 0.13 |
| N_issue_ | 16 | 16 | 16 | 52 | 52 | 52 |
| N_issue-year_ | 98 | 98 | 98 | 137 | 137 | 137 |
| N_individual_ | 2729 | 2729 | 2729 | 908 | 908 | 908 |
| N_obs_ | 23061 | 23061 | 23061 | 28851 | 28851 | 28851 |
| AIC | 19761.8 | 19739.6 | 19738.2 | 24140.8 | 24108.8 | 24101.1 |
| BIC | 19842.3 | 19828.1 | 19842.8 | 24223.5 | 24208.1 | 24216.9 |

*Note*. To measure media consumption we used the following items. In the GSS: “How often do you read the newspaper--every day, a few times a week, once a week, less than once a week, or never?” In the ANES: The maximum of “How many days in the past week did you read a daily newspaper?” and “How many days in the past week did you watch national news on TV?”

#

# Supplementary Table S5. Mixed effects logistic regressions of opinions among conservatives.

|  | ANES | | GSS | |
| --- | --- | --- | --- | --- |
|  | M0 | M1 | M0 | M1 |
| (Intercept) | −0.36 [−1.04, 0.32] | −0.35 [−1.03, 0.32] | 0.49 [0.07, 0.92] | 0.50 [0.07, 0.92] |
| Age | −0.07 [−0.09, −0.04] | −0.06 [−0.09, −0.04] | −0.31 [−0.36, −0.27] | −0.32 [−0.36, −0.27] |
| Education | 0.17 [0.14, 0.19] | 0.17 [0.15, 0.20] | 0.33 [0.28, 0.37] | 0.33 [0.28, 0.37] |
| Female | 0.00 [−0.05, 0.06] | 0.00 [−0.06, 0.05] | −0.08 [−0.17, 0.00] | −0.08 [−0.17, 0.00] |
| Black | 0.62 [0.50, 0.74] | 0.61 [0.50, 0.73] | 0.24 [0.09, 0.38] | 0.24 [0.09, 0.38] |
| Other | 0.20 [0.11, 0.29] | 0.20 [0.11, 0.29] | −0.04 [−0.27, 0.19] | −0.03 [−0.26, 0.20] |
| DF |  | −0.08 [−0.15, −0.01] |  | 0.00 [−0.06, 0.07] |
| σ_individual_ | 0.65 | 0.65 | 0.88 | 0.88 |
| σ_issue-year_ | 0.40 | 0.40 | 0.48 | 0.48 |
| σ_issue_ | 1.37 | 1.37 | 1.51 | 1.51 |
| σ_DFissue-slope_ |  | 0.13 |  | 0.17 |
| N_issue_ | 16 | 16 | 52 | 52 |
| N_issue-year_ | 120 | 120 | 137 | 137 |
| N_individual_ | 7316 | 7316 | 1168 | 1168 |
| N_obs_ | 65158 | 65158 | 38415 | 38415 |
| AIC | 63181.2 | 63054.4 | 40284.2 | 40225.3 |
| BIC | 63262.9 | 63163.4 | 40361.2 | 40328.0 |

*Note*. Model M0 is a null model of the probability among conservatives to hold a moral opinion with HVFL advantage, including only controls. Model M1 estimates the effect of discussion frequency on this probability.
